# Comparison of Titanium versus Resorbable Intramedullary Nailing in Pediatric Forearm Fractures

**DOI:** 10.3390/children11080942

**Published:** 2024-08-05

**Authors:** Ádám László Dávid, Flóra Mucsina, Eszter Antal, Anna Gabriella Lamberti, Aba Lőrincz, Gergő Józsa

**Affiliations:** 1 Division of Surgery, Traumatology, Urology and Otorhinolaryngology, Department of Pediatrics, Clinical Complex, University of Pécs, 7 József Attila Street, H7623 Pécs, Hungary; daaxaao.pte@pte.hu (Á.L.D.); lamberti.anna@pte.hu (A.G.L.); 2 Department of Thermophysiology, Institute for Translational Medicine, Medical School, University of Pécs, 12 Szigeti Street, H7624 Pécs, Hungary; aba.lorincz@gmail.com; 3 Department of Traumatology and Hand Surgery, Medical School, University of Pécs, 14 Ifjúság Street, H7624 Pécs, Hungary; flora.mucsina@aok.pte.hu; 4 Medical School, University of Pécs, 12 Szigeti Street, H7624 Pécs, Hungary; fcivf8@tr.pte.hu

**Keywords:** pediatric, forearm, fracture, poly-lactic-co-glycolic acid, resorbable, intramedullary nailing

## Abstract

Pediatric forearm fractures, particularly involving the shaft or diaphysis, are common injuries typically resulting from accidental trauma during various activities. Traditional treatment involves closed reduction and casting; however, surgical intervention may be necessary in certain cases. The gold standard surgical approach utilizes elastic stable intramedullary nailing (ESIN), but a newer technique uses bioabsorbable intramedullary nails made of poly(lactic-co-glycolic acid) (PLGA). This study aims to compare the outcomes of these two surgical methods in pediatric diaphyseal forearm fractures. We retrospectively reviewed 86 patients who underwent operative treatment due to the diaphyseal fractures of the forearm in the Surgical Division, Department of Pediatrics, Medical School, University of Pécs, Pécs, Hungary between 2018 and September 2022. The mean age was 9.48 (ranging from 4 to 17). A total of 41 patients underwent surgery with PLGA implants (RESIN technique), while 45 patients were treated with titanium elastic nails (ESIN technique). Various factors including patient demographics, injury mechanisms, fracture characteristics, and complications were assessed. Both groups showed similar gender distribution, with a majority of fractures occurring in boys (the male–female ratio was 31:10 in the PLGA group, while in the titanium elastic nailing (TEN) group, this ratio was 29:16, with no statistical difference between the groups (*p* > 0.005). The average age of the patients treated with PLGA implants (8.439 years) was lower compared to those treated with titanium nails (10.422 years). A statistically significant difference was found regarding the average age of the two groups (*p* = 0.0085). Left-sided injuries were more prevalent in both groups (59% of the cases in the PLGA group and 69% in the TEN group, with no statistically significant difference, *p* = 0.716), and fractures typically involved both the radius and ulna. This represents 93% of the cases in the PLGA group and 80% in the TEN group. Regarding the involvement of bones, we also did not find a statistically significant difference (*p* = 0.123). The mechanisms of injury predominantly involved indirect force, such as falls (30 cases in the PLGA group and 27 cases in the TEN group), and no statistically significant difference was found (*p* = 0.139) regarding the mechanism of the injury. Complication rates were lower in the PLGA group (7%) compared to the titanium group (20%). The treatment of pediatric diaphyseal forearm fractures using PLGA implants appears to be a viable alternative to traditional titanium implants. Advantages include no need for secondary surgery and associated cost savings and reduced complication rate and stress associated with anesthesia and surgery. Prospective randomized trials are warranted to further validate these findings and explore long-term outcomes.

## 1. Background

Among long-bone fractures in children, forearm fractures are the most common and make up approximately half of all childhood fractures. This translates to an annual prevalence of about 1.9% in the pediatric population [1,2]. Of forearm fractures, 20% involve the shaft, or the diaphysis of the bone [2]. Fracture incidence gradually increases in both genders up to the age of 11–12 years, then significantly decreases in girls while continuing to rise in boys. Beyond the age of 13, males experience twice as many forearm fractures as females [3].

The primary cause of these injuries is typically accidental trauma resulting from various sports or recreational activities. Specifically, team ball sports and wheeled activities such as cycling, roller skating, and skateboarding accounted for the majority of cases, comprising 42.4% and 34.9% of the incidents, respectively [4]. A study conducted in Wales in 1996 [4] also noted that 45% of the fractures that occurred in school settings took place on playgrounds. Among these cases, three-quarters of the injuries occurred while the child was running, and half of them resulted from falls on hard surfaces [4,5,6,7]. In the context of distal radial fractures, the typical mechanism of injury is a fall on an outstretched arm, often abbreviated as FOOSH. Forearm shaft fractures often occur also this way or due to a direct hit to the forearm [8,9,10].

Most childhood forearm fractures can be successfully treated conservatively using closed reduction and cast immobilization. Closed, non-displaced, or minimally displaced fractures of the radius and ulna resulting from low-energy trauma can be managed with a long arm (above-elbow) cast. The duration of immobilization typically ranges from 3 to 6 weeks depending on the child’s age [7]. Opinions may vary on when surgical treatment is necessary. In addressing these varied opinions, Bašković et al. conducted a systematic search in the PubMed database. They gathered articles that clearly address the question of when surgical treatment is unnecessary, as it would only lead to higher costs and increased complication rates [11]. Absolute surgical indications include first-, second-, and third-degree open fractures, especially if the fracture is associated with neurovascular compromise or compartment syndrome [12]. Relative surgical indications encompass irreducible fractures or fractures that remain unstable even after reduction, such as complete oblique or comminuted fractures. Surgical intervention is also recommended for the treatment of redislocated fractures following unsuccessful conservative management [7]. Currently, the gold standard operative management for pediatric diaphyseal forearm fractures is ESIN. The titanium elastic nail (TEN) to be inserted into the ulna is most commonly from the proximal end of the bone, while the insertion of the radial intramedullary nail is performed from the distal end [13]. The titanium implant is recommended to be removed between 6 and 12 months after the primary treatment. The removal is associated with risks related to repeated anesthesia and surgery, causing physical and psychological discomfort and burden for both patients and parents, as well as adding extra costs to the healthcare system [12].

In a similar surgical technique, we can use bioabsorbable implants instead of titanium to stabilize the fracture. These devices are made of biocompatible polymers and their degradation primarily occurs through hydrolysis [14]. In the cases of pediatric forearm fractures, bioabsorbable intramedullary nails made of PLGA are used, which is a slowly resorbable polymer. As a result, during the degradation of the nail, the load is gradually transferred to the healing bone, thereby promoting its remodeling. Bioabsorbable intramedullary nails are introduced similarly to the already mentioned traditional ESIN technique, with some differences due to the different materials of the implant. Postoperative immobilization with a cast is recommended for bioabsorbable implants due to their lesser stability. Cast options include a long arm cast for two weeks followed by a short arm cast for two weeks or a long semicircular arm cast with volar support for four weeks [14,15,16].

This retrospective study aims to compare these two surgical techniques in children who were treated for diaphyseal forearm fractures. In this retrospective study, we wanted to compare these two surgical techniques, hypothesizing that the novel method can be a sufficient alternative to the gold standard ESIN method.

## 2. Patients and Methods

### 2.1. Study Design

Data from the patients of the Surgical Division, Department of Pediatrics, Medical School, University of Pécs, Pécs, Hungary, were examined between 2018 and September 2022. We retrospectively reviewed 86 patients who underwent operative treatment due to the diaphyseal fractures of the forearm. Surgical treatment was performed with an intramedullary nail, which was either titanium or an implant made of absorbable biopolymer, PLGA. The patients treated with the resorbable implant were from the period between March 2021 and September 2022, while the children operated with the titanium elastic nail were from the period between 2018 and 2022. We examined the children admitted to our clinic with diaphyseal forearm fractures requiring surgical intervention. This is a prospective view on the resorbable implants, meaning that after March 2021, we treated children with resorbable implants in 41 cases. For comparison, we selected a similar number of cases from the children treated with titanium implants. This represents the 45 cases we previously operated on. No selection was made among the patients included in the study.

### 2.2. Participants

The inclusion criteria were as follows: (1) an open growth plate, (2) an isolated radius or forearm diaphyseal (middle part) fracture with both affected bones, and (3) a fracture requiring surgical treatment. The exclusion criteria were as follows: (1) a closed growth plate or (2) associated vascular or nerve injury with the fracture.

All the procedures were carried out while the patients were under general anesthesia and guided by the control of a C-arm image intensifier. A single-shot antibiotic prophylaxis was routinely applied in both groups. All the operations were conducted by pediatric surgeons with experience in pediatric traumatology. The fixation of the fracture was carried out with the traditional, gold standard method called elastic stable intramedullary nailing in the cases of 45 children. In the cases of 41 children, a novel operative technique with a resorbable intramedullary implant (Activa IM-Nails™, Bioretec^®^, Tampere, Finland) was performed.

### 2.3. Evaluated Endpoints

Several factors were studied during data processing, including the sex and age of the patients, along with the mechanism and location of the injury. We also examined which arm was injured and whether the radius, the ulna, or both bones were fractured. In addition, we investigated the diameter of the inserted resorbable or metal intramedullary nail, and what complications arose from the implantation of the devices.

### 2.4. Operative Technique with Elastic Stable Intramedullary Nail (ESIN)

**Figure 1:**
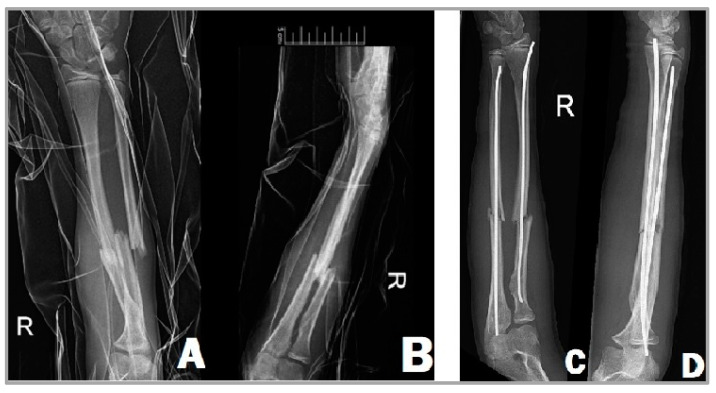
X-ray images of a 13-year-old girl’s right forearm from anteroposterior (**A**) and lateral (**B**) views before surgery, showing the complete fractures of the radius and ulna. After the surgery (**C**,**D**), the two TENs fixate the fracture in a good position.

The titanium elastic nail to be inserted into the ulna is most commonly from the proximal end of the bone, while the insertion of the radial intramedullary nail is carried out from the distal end of the radial side. The minimally bent tip of these implants makes reduction easier. Insertion is performed under the control of intraoperative imaging with C-arm fluoroscopy. Regarding the length and the diameter of the implant, it can be stated that it is recommended to insert a nail that fills two-thirds of the medullary canal. The usage of a thinner nail can lead to the instability of the reduction and delayed fracture healing [17,18]. First, we have to attempt the closed reduction of the fracture, but if this is unsuccessful, we can perform a percutaneous reduction of the fracture with a guide wire inserted into the fracture site [19]. Once the desired position is achieved, we insert the elastic nails into both the radius and the ulna (Figure 1). In children operated with ESIN, there is usually no need for plaster immobilization and their emission from the hospital can take place on the first or second postoperative day.

### 2.5. Operative Method with Resorbable Intramedullary Nail (RESIN)

**Figure 2:**
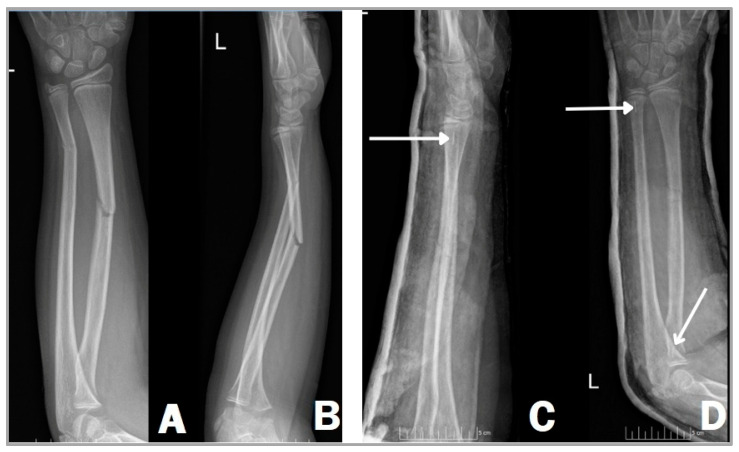
Right forearm shaft fracture of an 11-year-old girl, showing complete fractures in both radius and ulna. Anteroposterior (**A**) and lateral (**B**) X-rays before and after surgery (**C**,**D**). Two Activa IM-Nails™ fixate the fracture in a good position. The white arrows show the radiopaque tricalcium phosphate (β-TCP) tips (**C**,**D**).

In a similar surgical technique, we can use bioabsorbable implants instead of titanium to stabilize the fracture (Figure 2). The position of the patient and the preparation are no different from the gold standard method, utilizing a supine position on a standard operating table with a radiolucent arm board [20,21]. A C-arm image intensifier is used also during this technique; however, only the radiopaque tricalcium phosphate (β-TCP) tip is visible on the film, not the entire nail as with TENs (Figure 2C,D), due to the PLGA material being radiolucent. After the reduction of the fracture, unlike the ESIN procedure, the medullary canal should be prepared with an implant-specific dilator tool when using a bioabsorbable intramedullary nail. This procedure is carried out in order to reduce the chances of implant fracture during insertion against resistance. Dilator tools are employed to create a suitable area within the canal for accommodating the implant [14,15,16]. After that, the inserted steel dilator alloys are removed and replaced by biodegradable nails (Activa IM-Nails™). This can only be achieved when both bones of the forearm are securely stabilized by the aforementioned dilators and it is also essential to remove the dilator from one bone at a time. Moreover, it is important to remove the metallic instrument from the second bone only when the resorbable implant placed in the first bone is in position and adequately stabilizes the fracture to reduce the risk of secondary displacement [22].

During the placement of the resorbable implant, high-energy movements should be avoided to prevent implant breakage. The implants are typically easily inserted under continuous, low-energy pressure. If there is any resistance, it is important not to force the insertion. In such cases, it is recommended to perform dilation again using a thicker dilator or TEN. After insertion, the protruding proximal end of the implant should be cut off because there is no need for removal later, thereby preventing potential complications such as skin irritation [22].

Another difference from traditional techniques is that post-surgery, it is necessary to immobilize the affected limb for four weeks. Initially, this involves an above-elbow cast, which may later be replaced with a short cast or brace if deemed appropriate by the surgeon during the control examination [22].

### 2.6. Statistical Analysis

Descriptive statistics for all the endpoints were calculated, including the means, standard deviations (SD), medians, interquartile ranges (IQR), 25th percentiles (IQR25), 75th percentiles (IQR75), counts, and ranges. For discrete outcomes, the relative and absolute distributions were also evaluated. Statistical comparisons were performed using Chi-square tests for categorical variables and the Mann–Whitney U test for continuous outcomes. Due to the relatively small population, Shapiro–Wilk tests were utilized to assess the distribution normality of continuous variables. The analyses were conducted using Python 3.9.19 (Python Software Foundation, Wilmington, DE, USA) with the NumPy, pandas, and scikit-learn libraries for data manipulation and evaluation. A *p*-value of ≤0.05 was considered indicative of statistical significance.

## 3. Results

Out of the 41 children who underwent surgery with resorbable implants, 31 were boys (75.61%) and 10 were girls (24.39%). In the group treated with titanium implants, we investigated 45 cases of forearm fractures which included 29 boys (64.44%) and 16 girls (35.56%). In both groups, a majority of the cases were observed in boys. No statistical difference was found between the groups regarding sex (see Table 1, *p* > 0.005).

The youngest among the children treated for forearm diaphysis fracture was four years old, while the oldest was 15. The 4-year-old patient required surgery due to a fracture that involved significant displacement and considerable misalignment, resulting in serious instability. The average age of children treated with resorbable implants was 8.44 years (ranging from 4 to 14 years), whereas for those treated with metal nails, this value was 10.42 years (range: 6–15 years). A statistically significant difference was found regarding the average age of the two groups (see Table 1). The use of TEN is more common in older age groups, which is understandable considering that adolescents often present with injuries when they are nearly adult-sized. In such cases, it is more prudent to consider the application of the gold standard procedure. This may account for the observed statistically significant difference. Based on the data, it can be stated that boys of all age groups experienced forearm fractures, while among girls, it was more prevalent at a younger age, up to 11 years. Although the significant increase after 11 years, as reported in the international literature, is not evident in our data for boys, the decrease observed in girls is clearly visible in our table (Table 1) [1,2]. However, this difference could also be attributed to the low number of cases we examined.

We analyzed whether children more commonly experienced fractures in the right or left forearm diaphysis. Based on our evaluated data, we can conclude that both the patients treated with resorbable implants (59%) and the patients treated with titanium elastic nails (69%) exhibited a higher number of left-sided injuries (Table 1). However, regarding the laterality of the injury, this difference is not statistically significant (see Table 1, *p* = 0.716).

We can ascertain that in the groups we examined, dominantly combined radius and ulna fractures were observed (80% in the TEN group and 93% in the resorbable group). Among the children treated with resorbable implants, in three cases, only one of the bones was fractured (7%), while in those treated with TENs, this number was nine (20%) (Table 1). Regarding the involvement of bones, we also did not find a statistically significant difference (Table 1, *p* = 0.123).

**Figure 3:**
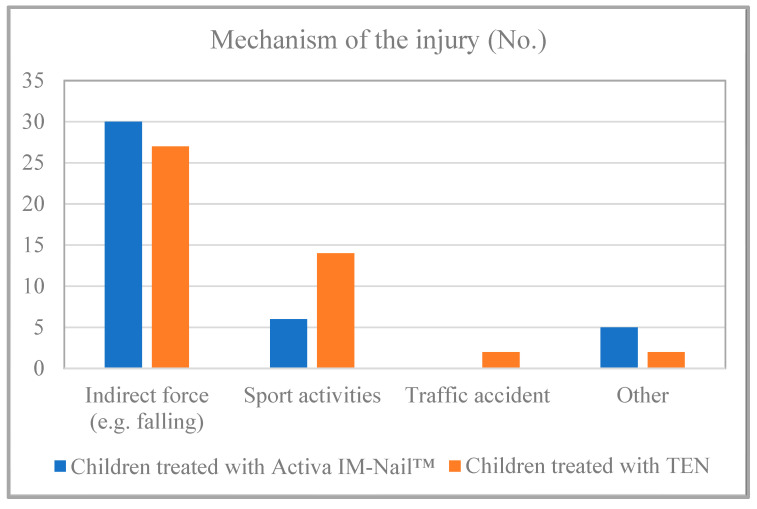
Etiology of the fractures.

Based on the data from the study’s examined children, it can be affirmed that no statistically significant difference was found (*p* = 0.139) regarding the mechanism of the injury. Approximately three-quarters (73%) of the resorbable group and nearly two-thirds (60%) of the TEN group sustained injuries due to indirect force, such as a fall. Injuries during sports activities also suggest the possibility of indirect force in some cases. Forearm fractures resulting from high-energy accidents occurred in a smaller number of cases (Figure 3).

**Figure 4:**
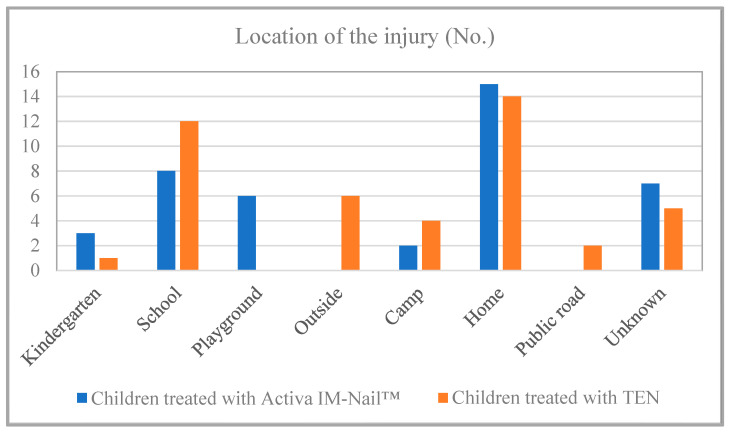
Location of the accident occurring.

Observing the location of the injuries, it can be noted that most forearm fractures occurred at home (31.11% in the TEN group and 36.59% in the resorbable group), while the number of fractures occurring at school (26.67%) is approaching this figure among the children treated with TEN (Figure 4). There is a significant difference between playground and outdoor hued by implant type with a corrected *p*-value of 0.0218. The analysis with Fisher’s Exact Test and Holm–Bonferroni correction found a significant difference between the subgroups for injury place.

In approximately two-thirds of the patients treated with absorbable implants, specifically in 25 cases (61%), a medullary nail with a diameter of 2.7 mm was used. A similar ratio was observed among the children treated with titanium elastic nails, with also 25 cases (56%) receiving an implant with a diameter of 2.5 mm.

### Complications

Among the children treated with absorbable implants, complications occurred in three cases (7%). In two cases (5%), redislocation occurred, ranging between 0 and 10°, and did not require additional surgery. In one case (2%), there was an implant-related complication where the absorbable nail split during insertion due to the sharp fracture surface injury. Among the children treated with TENs, complications occurred in eleven instances (20%). In five cases (9%), irritation symptoms developed, and in two cases (4%), skin perforation occurred, caused by the end of the implant. In the cases of skin perforation, the elastic nail was trimmed, while early metal removal was performed due to irritation symptoms. Three patients (5%) experienced injury to the sensory branch of the radial nerve. In one child (2%), redislocation occurred with a fracture angle between 0 and 10°, which did not require surgical correction. Ninety-three percent of the children treated with absorbable implants and 80% of those treated with TENs recovered without complications.

## 4. Discussion

In this study, we aimed to compare the two previously mentioned operative techniques, ESIN and RESIN, hypothesizing that the new method can be a sufficient alternative to the gold standard ESIN method. Regarding our results, no statistical difference was found between the groups regarding sex (*p* > 0.005). It can be stated that there is a predominance of boys in both groups (75.61% in the absorbable group and 64.44% in the TEN group), which aligns with the ratios found in the international literature [2,7]. A statistically significant difference was found regarding the average age of the two groups (*p* = 0.0085). This statistically significant difference unfortunately does not align with the results of a similar Finnish study; however, it is important to note that Korhonen et al. worked with a significantly smaller sample size [23]. The use of TEN is more common in older age groups, which is understandable considering that adolescents often present with injuries when they are nearly adult-sized. In such cases, it is more prudent to consider the application of the gold standard procedure. This may account for the observed statistically significant difference. Our data indicate that boys of all age groups experienced forearm fractures, whereas among girls, these fractures were more prevalent up to the age of 11. Although the significant increase in fractures after 11 years, as noted in the international literature, is not evident in our data for boys, the decrease observed in girls is clearly shown in Table 1 [1,2]. In the groups we examined, dominantly combined radius and ulna fractures were observed (80% in the TEN group and 93% in the resorbable group). Among the children treated with resorbable implants, in three cases, only one of the bones was fractured (7%), while in those treated with TENs, this number was nine (20%). These ratios correspond to those reported in a 2018 Finnish study in which Korhonen et al. examined the operative treatment of pediatric diaphyseal forearm fractures using ESIN or RESIN techniques in a small sample study, with the primary focus on range of motion [23]. Regarding the involvement of bones, we also did not find a statistically significant difference (Table 1, *p* = 0.123). Based on the data from the children examined in the study, it can be affirmed that approximately three-quarters (73%) of those with forearm fractures treated with resorbable implants and nearly two-thirds (60%) of those managed with TENs sustained their injuries due to indirect forces, such as falls. This high percentage is consistent with findings reported in international studies [1,2]. Regarding the mechanism of the injury, no statistically significant difference was found (*p* = 0.139). Approximately three-quarters (73%) of the resorbable group and nearly two-thirds (60%) of the TEN group sustained injuries due to indirect force, such as a fall. These ratios are similar to the results of international studies that work with large sample sizes and focus on epidemiological factors [1,2]. As can be seen in our results, there were no significant differences between the methods; however, in our study, more complications (11 cases) occurred in the children treated with titanium nails. This number in the case of the children treated with bioabsorbable implants was only three. One of the complications can be associated with the material of the implant when the PLGA implant splits during insertion. However, it was due to the sharp fracture surface injury, which can be hardly seen. It is important, however, to note that the difference in complication rates may also arise from the fact that the RESIN technique involves a more gentle approach, resulting in less trauma of the tissue during surgery. Furthermore, the postoperative immobilization applied also leads to fewer soft tissue and infectious complications. One of the biggest disadvantages of PLGA implants is that they are hardly visible under fluoroscopy and because of that, it requires experienced surgeons to operate. Currently, there are intramedullary PLGA nails with bio-labeled ends available, offering a potential solution to this issue. Alternatively, unfavorable soft tissue reactions could be provoked by the material; however, neither in our study nor in the literature have we encountered that. PLGA undergoes gradual hydrolysis and is steadily eliminated from bone tissue over the course of several years [23,24,25]. Another potential issue might involve the onset of an infectious complication. While none of our patients have experienced microbial overcolonization, it is crucial to be ready for such an occurrence, because a solution could involve the complete removal of the nails and thorough cleansing of the medullary cavity. They can be challenging to extract due to post-insertion swelling; therefore, drilling may be necessary. Additionally, it is worth noting that deep septic complications following intramedullary nailing in children are rare in the literature, and there were no publications documenting such occurrences with PLGA implants [25].

### Limitations

The biggest constraint of this study is its retrospective nature and small population. The follow-up duration was also short, and because of that, complications may occur later on. Additional limitations of the article include the lack of randomization and blinding, as well as the absence of a predefined protocol, which consequently increases the risk of bias. Furthermore, there is no cost-effectiveness analysis, nor are pain and severity reported. The use of higher-level imaging techniques (such as MRI) could also have been beneficial in the study. Further studies are recommended to solve these problems. Despite these limitations, we believe that our results can shed light on the potential of absorbable implants as a suitable alternative to the currently used gold standard ESIN technique in the treatment of pediatric forearm shaft fractures.

## 5. Conclusions

Based on our results and the literature data, it can be stated that pediatric forearm shaft fracture treatment using absorbable implants could be a suitable alternative to treatment with metallic devices. An advantage of biopolymers is that there is no need for another surgery to remove the implanted device. Consequently, this not only reduces the chance of complications but also minimizes psychological stress on children. Since there is no need for a second operation, significant cost savings can be achieved. However, these promising initial findings should be validated through prospective randomized trials.

## Figures and Tables

**Table 1:** Characteristics of the patients enrolled in the study.

Characteristics	Titanium Elastic Nailing Group (*n* = 45)	Bioresorbable Group (*n* = 41)	*p*-Values
Average age; years	10.422	8.439	0.0085 ^1^
Sex ratio (Number of patients) (male–female)	1.8125 (29:16)	3.1 (31:10)	0.430 ^2^
**Injured upper limb side (No., %)**			
Right	14 (31)	17 (41)	0.716 ^2^
Left	31 (69)	24 (59)	
**Affected bone (No., %)**			0.123 ^2^
Radius (isolated)	9 (20)	2 (5)	
Ulna (isolated)	0 (0)	1 (2)	
Both bones	36 (80)	38 (93)	

^1^ Mann–Whitney U test. ^2^ Chi-squared test.

## Data Availability

All data are contained within the article.
